# Supplementary material for: Connexin 32 constrains a mesenchymal-like switch in differentiated urothelium and luminal cancers
Source: Life Sci Alliance. 2026 Feb 17;9(5):e202503427. doi: 10.26508/lsa.202503427 (PMC12912911; doi:10.26508/lsa.202503427)
Supplement: Supplementary file 1 [file LSA-2025-03427_SdataF1.pdf]

Labelled with anti-**Cx32** antibody - predicted molecular weights at 32 kD (monomer) and 54 kD (dimer)

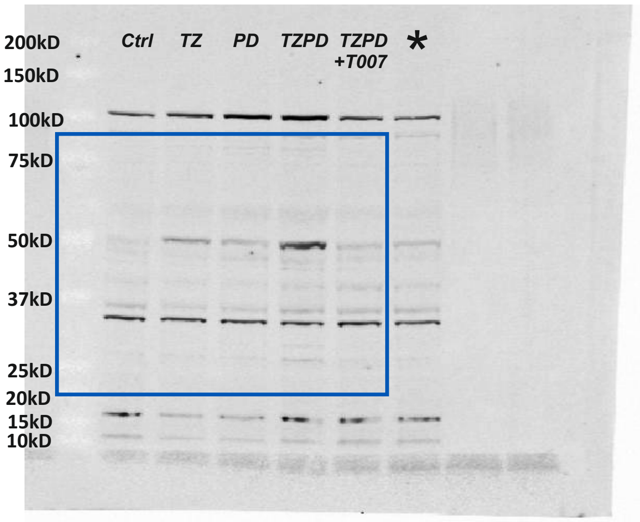

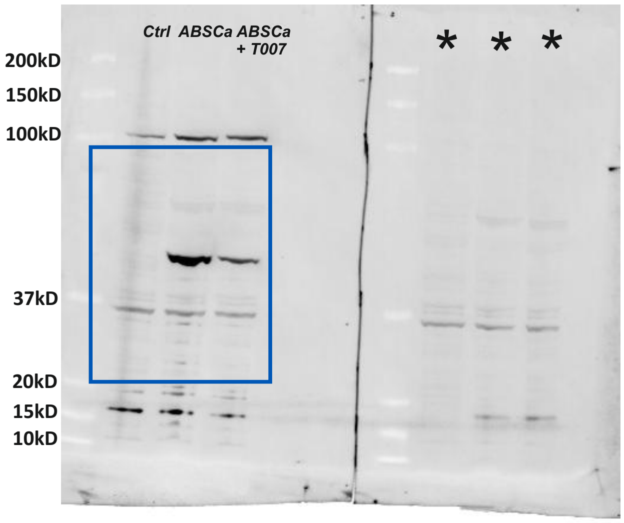

Labelled with anti- **β-actin** antibody - predicted molecular weight = 42 kD

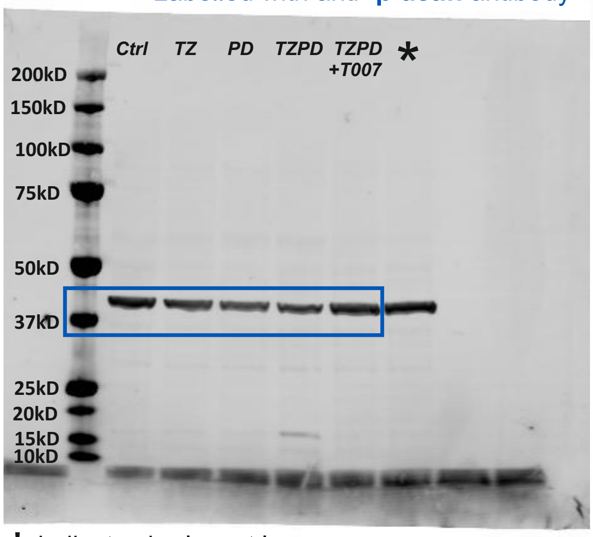

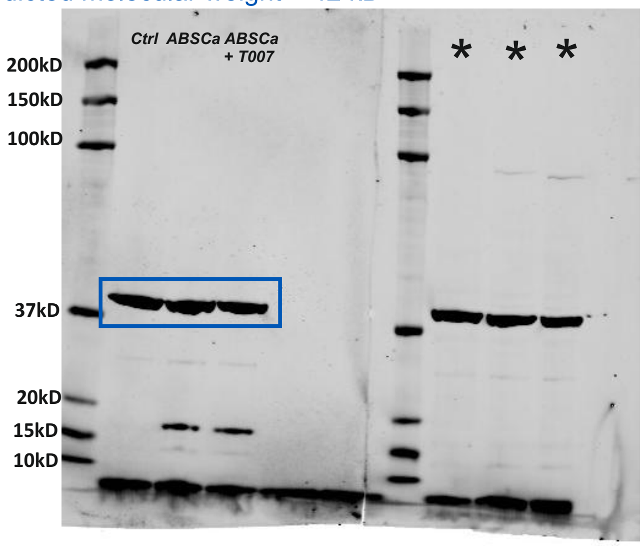

* indicates irrelevant lanes

Blue boxes indicate approximate cropped regions for final figure
Note two different ladders were used (Biorad Precision Plus All Blue, or Dual Colour) - molecular weights are marked
