# Supplementary material for: Connexin 32 constrains a mesenchymal-like switch in differentiated urothelium and luminal cancers
Source: Life Sci Alliance. 2026 Feb 17;9(5):e202503427. doi: 10.26508/lsa.202503427 (PMC12912911; doi:10.26508/lsa.202503427)
Supplement: Supplementary file 2 [file LSA-2025-03427_SdataFS1.pdf]

Labelled with anti-**Cx32** antibody - predicted molecular weights at 32 kD (monomer) and 54 kD (dimer)

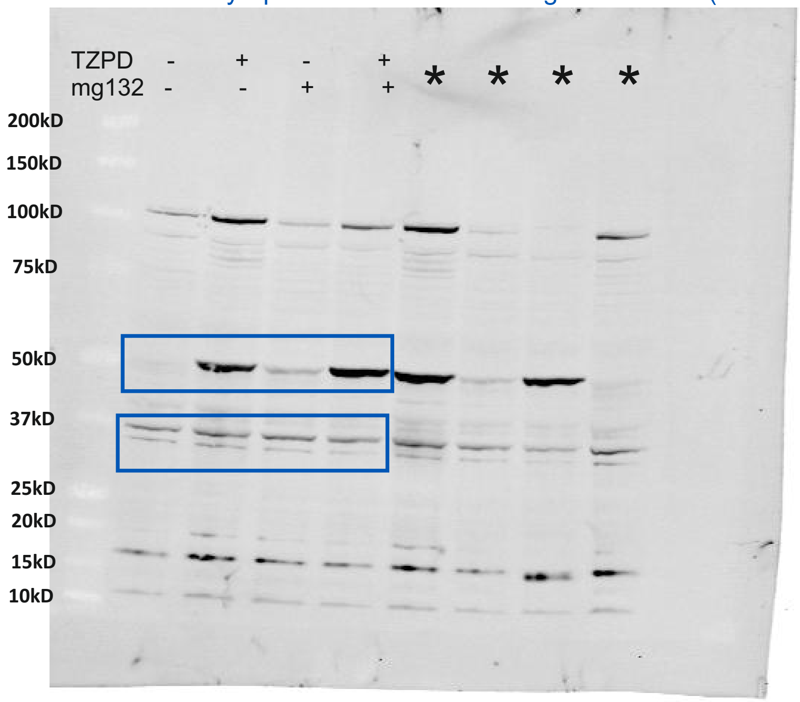

Labelled with anti- **β-actin** antibody - predicted molecular weight = 42 kD
and anti-**claudin 4** antibody - predicted molecular weight = 22 kD

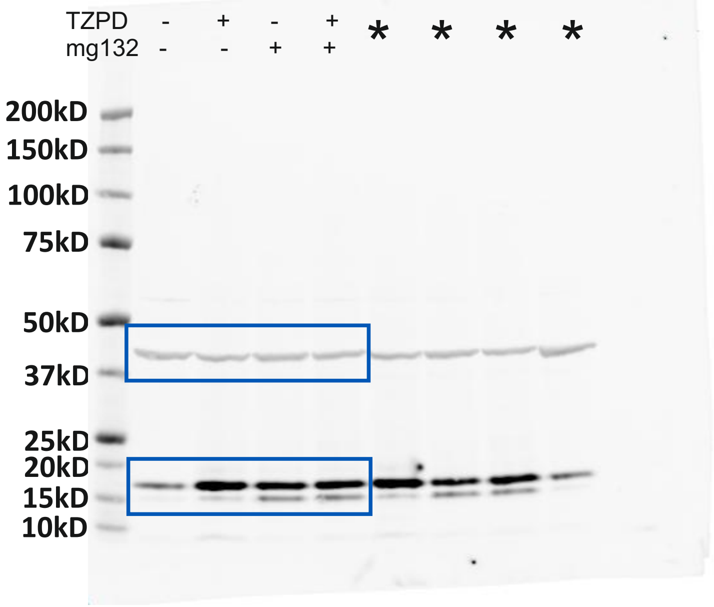

* indicates irrelevant lanes

Blue boxes indicate approximate cropped regions for final figure
Note All Blue ladder doesn't fluoresce well in the 800nm channel (used for Cx32 blot)
molecular weights are marked
