# Supplementary material for: Connexin 32 constrains a mesenchymal-like switch in differentiated urothelium and luminal cancers
Source: Life Sci Alliance. 2026 Feb 17;9(5):e202503427. doi: 10.26508/lsa.202503427 (PMC12912911; doi:10.26508/lsa.202503427)
Supplement: Supplementary file 3 [file LSA-2025-03427_SdataF2.pdf]

Labelled with anti- **β-actin** antibody - predicted molecular weight = 42 kD

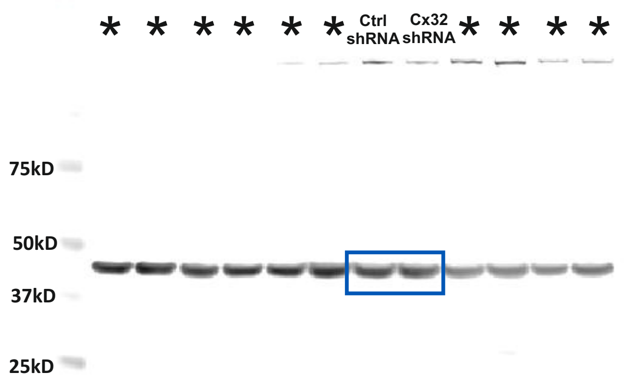

Labelled with anti-**Cx32** antibody - 54 kD (dimer) is marked

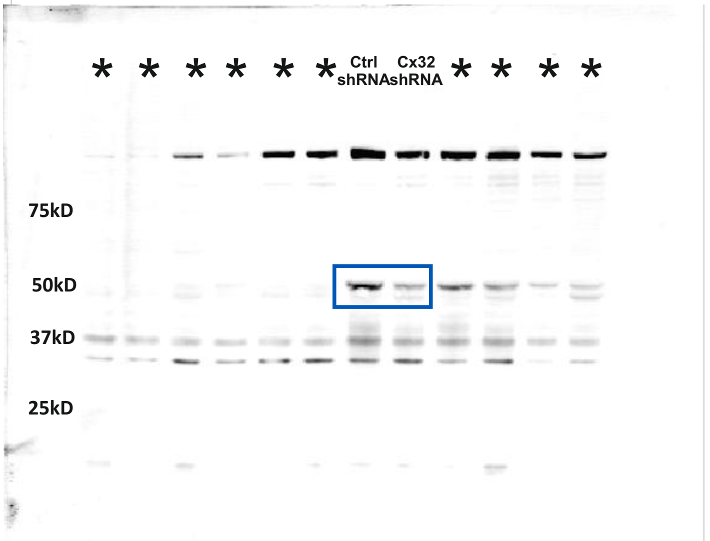

Labelled with anti- **β-actin** antibody - predicted molecular weight = 42 kD

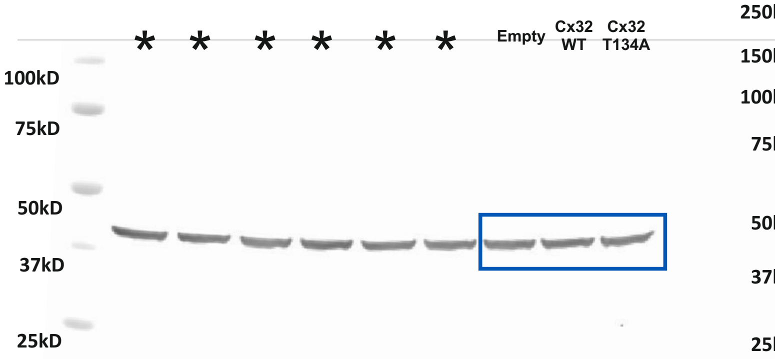

Labelled with anti-**Cx32** antibody - 54 kD (dimer) is marked

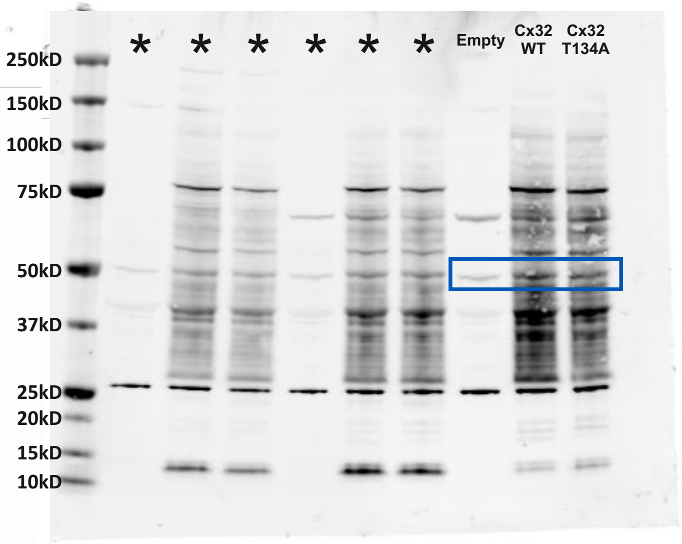

* indicates irrelevant lanes

Blue boxes indicate approximate cropped regions for final figure
Note Biorad Precision Plus ladder does not fluoresce in the 800nm channel (used for the upper Cx32 blot), but the upper two scans are on the same membrane and were aligned to identify molecular weights
