# Supplementary material for: Connexin 32 constrains a mesenchymal-like switch in differentiated urothelium and luminal cancers
Source: Life Sci Alliance. 2026 Feb 17;9(5):e202503427. doi: 10.26508/lsa.202503427 (PMC12912911; doi:10.26508/lsa.202503427)
Supplement: Supplementary file 5 [file LSA-2025-03427_SdataF5.pdf]

Labelled with anti-Slug antibody
Predicted molecular weight = 30kD

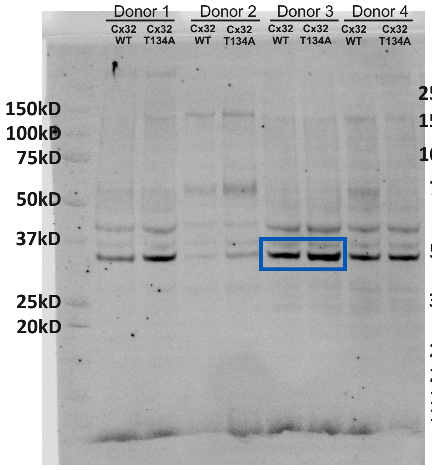

Labelled with anti-P-Cadherin antibody
Predicted molecular weight = 90-120kD

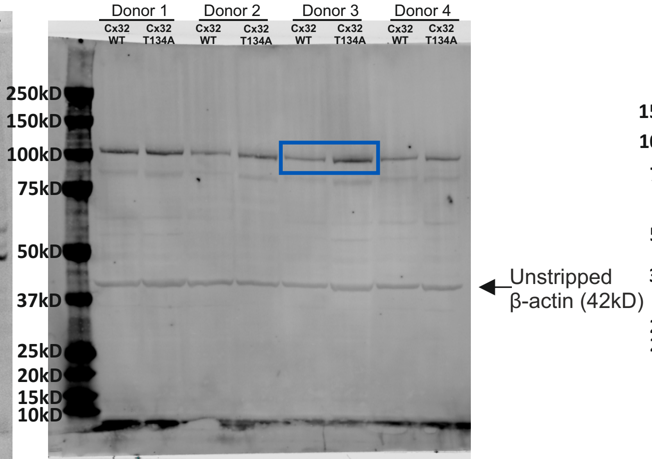

Labelled with anti-vimentin antibody
Predicted molecular weight = 54kD

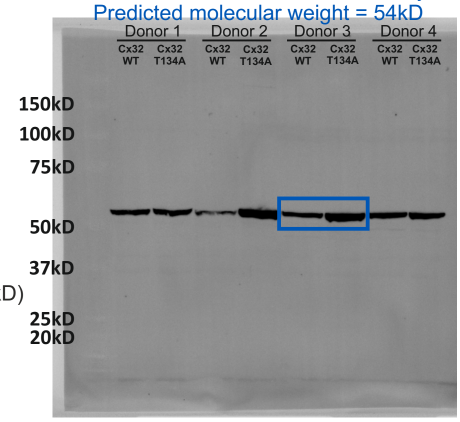

Labelled with anti-E-cadherin antibody
Predicted molecular weight = 120kD

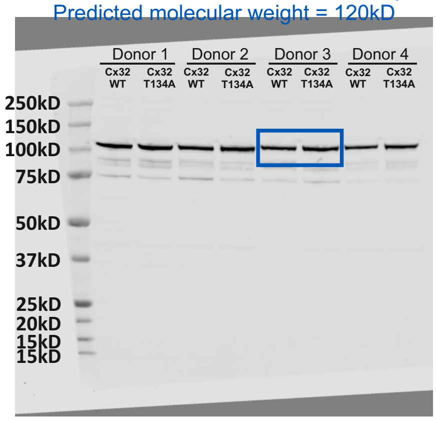

Labelled with anti-pSMAD1/3/5 antibody
Predicted molecular weights = 50 and 52-54kD

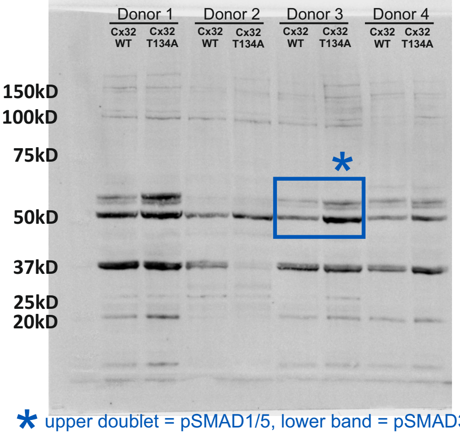

* upper doublet = pSMAD1/5, lower band = pSMAD3

Labelled with anti-Cox2 antibody
Predicted molecular weights = 72kD

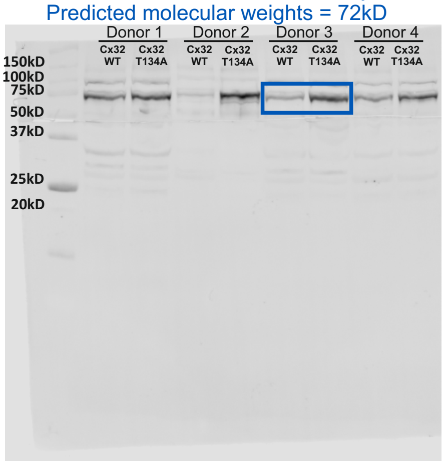

Labelled with anti-total SMAD3 antibody
Predicted molecular weight = 42kD

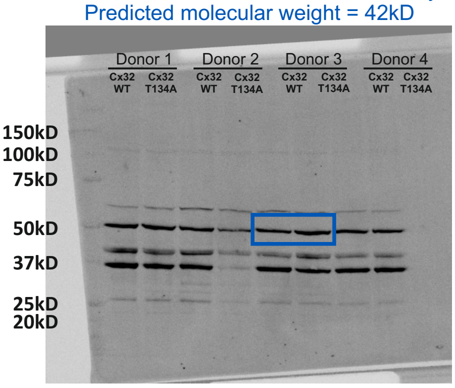

Labelled with anti-claudin 3 antibody
Predicted molecular weight = 18kD

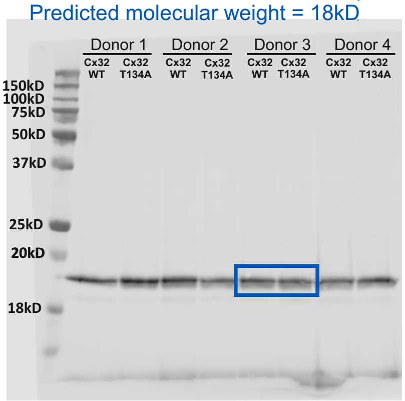

Labelled with anti-beta-actin antibody
Predicted molecular weight = 42kD

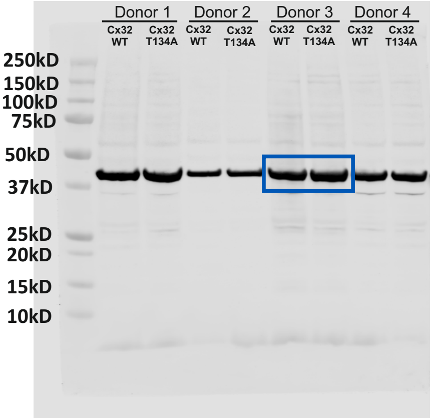

Blue boxes indicate approximate cropped regions for final figure
Note Biorad Precision Plus ladder does not fluoresce in the 800nm channel (used for SMAD, slug and vimentin)
